# Improved cardiac arterial spin labelling in the mouse heart by optimisation of acquisition and analysis

**DOI:** 10.1186/1532-429X-13-S1-P56

**Published:** 2011-02-02

**Authors:** Adrienne E Campbell, Anthony N Price, Jack A Wells, Mark F Lythgoe, Roger J Ordidge

**Affiliations:** 1 Centre for Advanced Biomedical Imaging, University College London, London, UK; 2 Robert Steiner MRI Unit, Imperial College London, London, UK; 3 Department of Medical Physics and Bioengineering, University College London, London, UK

## Objective

In this study, a time-efficient segmented k-space arterial spin labelling (ASL) sequence is implemented for the mouse heart, and data quality is improved using a data logger for additional gating and retrospective analysis.

## Background

Myocardial perfusion is an indicator of cardiac health and an important parameter in detection and follow-up of cardiac disease. Myocardial perfusion can be measured in the mouse non-invasively with MRI using ASL (eg. [1], [2]).

## Methods

Perfusion maps are generated by pixel-wise comparison of T_1_ maps from slice-selective and global inversion recoveries. T_1_ was quantified using ECG-gated Look-Locker approach [3], with 4 lines of k-space acquired every heartbeat to speed up acquisition by approximately four times compared with previous approaches (eg. [2],[3]).

A data logger (CED) was used to record ECG, RF and respiration events and was programmed to gate on each ECG peak detected as well as to interpolate peaks missed by physiological monitoring software. Using the information recorded, lines of k-space were binned as ‘acquired during respiration’ or not. Images with many lines or centre lines of k-space acquired during respiration were rejected.

ASL data were acquired on a 9.4T Varian system with 35mm-long volume RF coil (Rapid) (TE=1.18ms, flip=5°, resolution=200μm, slice thickness=1.5mm) and image analysis was performed in Matlab.

## Results

**Figure 1:**
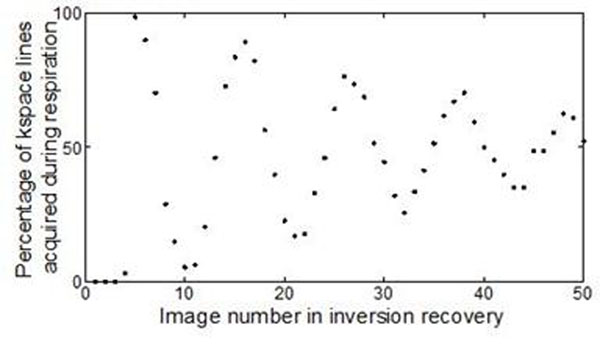
Percentage of k-space lines (for 128x128 matrix) which are acquired during respiration throughout inversion recovery.

It was found that an average of 2 ECG peaks per inversion recovery were missed by the physiological monitoring software, compared to none using the data logger script. The percentage of k-space lines acquired during respiration for images in an inversion recovery is displayed in Figure1. The pattern of Figure1 is generated by the de-phasing of respiration and acquisition with changing respiration rate. This manifests as blurring to the final images of the inversion recovery curve.

**Figure 2:**
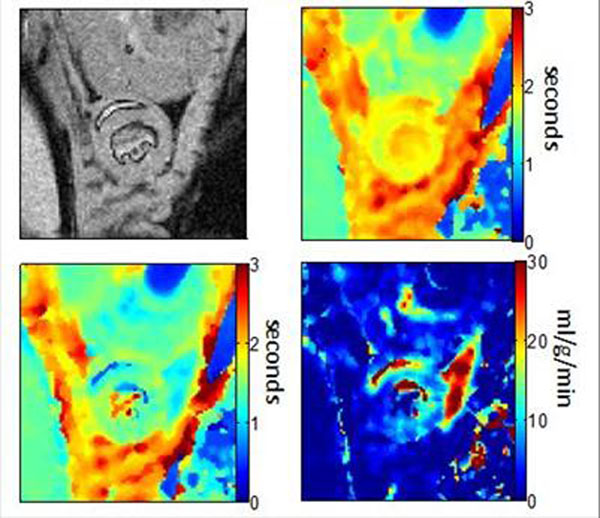
Example ASL data set from one mouse (heart rate = 550 bpm). a) Anatomical image, T1 map from b) global inversion recovery and c) slice-selective inversion recovery, and d) resulting perfusion map, with values greater than 30ml/g/min clipped.

Figure2 presents an example ASL data set. The mean perfusion in the myocardium is estimated to be 7.0±3.3ml/g/min, which is consistent with literature values. Anatomical image quality is not compromised using this segmented k-space method and T_1_ maps appear to have less respiration corruption.

## Conclusions

This study presents a time-efficient acquisition of cardiac ASL data in the mouse, with improved data quality using a data logger to eliminate missed ECG triggers and to introduce objective criteria for rejection of respiration-corrupted images.
